# Cardiovascular risk management in patients with active Ankylosing Spondylitis: a detailed evaluation

**DOI:** 10.1186/s12891-015-0532-3

**Published:** 2015-04-09

**Authors:** Sjoerd C Heslinga, Inge A Van den Oever, Alper M Van Sijl, Mike J Peters, Irene E Van der Horst-Bruinsma, Yvo M Smulders, Michael T Nurmohamed

**Affiliations:** Department of Rheumatology, Reade, Amsterdam, Netherlands; Department of Rheumatology, VU University Medical Centre, Amsterdam, Netherlands; Department of Internal Medicine, VU University Medical Centre, Amsterdam, Netherlands

**Keywords:** Ankylosing Spondylitis, Risk factors, Hypertension, Smoking, Treatment

## Abstract

**Background:**

Ankylosing spondylitis (AS) is an inflammatory disease with documented elevated cardiovascular (CV) risk due to systemic inflammation and a higher prevalence of CV risk factors. CV risk management (CV-RM) could be an effective method to reduce CV mortality and morbidity in AS patients. We assessed CV risk and evaluated guideline adherence according to the Dutch CV-RM guideline.

**Methods:**

This study was conducted with a cohort of consecutive AS patients eligible for treatment with a tumor necrosis factor (TNF) -α inhibitor. Data from the Dutch National Institute for Public Health and Environment was used to compare the prevalence of CV risk factors in AS patients with the Dutch background population.

**Results:**

In total, 254 consecutive AS patients were included. The prevalences of hypertension (41% vs 31%) and smoking (43% vs 27%) were substantially higher in AS patients as compared to the general Dutch background population. Of 138 AS patients older than 40 years the 10-years CV risk could be calculated. Fifty-one of these 138 patients (37%) had an indication for CV risk treatment. CV risk treatment was initiated in 42 of the 51 (82%), however, in only 12 of the 51 (24%) patients treatment targets for either hypertension or hypercholesterolemia were reached.

**Conclusion:**

The increased rates of hypertension and smoking illustrate the importance of CV-RM in AS patients. Although the majority of all AS patients eligible for CV-RM received CV risk medication, CV-RM remains a challenge for treating physicians, as treatment targets were not achieved in three-quarter of the eligible patients.

## Background

Ankylosing spondylitis (AS) is an autoimmune inflammatory disease associated with a decreased overall life expectancy compared to the general population [1,2]. Studies investigating mortality in AS show that the most common causes of death are of circulatory origin [3-5]. This increased cardiovascular (CV) mortality and morbidity is caused by multiple factors. Chronic inflammation acts independently or synergistically with traditional risk factors in the pathogenesis of atherosclerosis [3]. Obviously traditional CV risk factors, such as hypertension, hypercholesterolemia, and smoking remain important and modifiable contributors. Until now, it is still unclear which CV risk factors are more prevalent in the AS population compared to the general population, as studies on this subject and their results are heterogeneous [4-6].

Early identification and optimal CV risk management are mandatory to reduce the CV risk in AS patients. Worldwide, various CV risk management (CV-RM) guidelines with different algorithms are available [7,8]. Most of these algorithms do not include inflammation. The EULAR CV-RM guideline considers AS to be an important CV risk factor, but unlike rheumatoid arthritis (RA), does not suggest modification of CV risk calculation for this group, as epidemiological evidence supporting an increased CV risk is less evident in AS [9]. In recent years, however, accumulating evidence for the increased CV risk in AS is emerging [10-12]. So, similarly as in RA, one may consider multiplying the derived CV risk estimate by 1.5 or adding 15 years to the actual age of an AS patient when calculating CV risk for a better CV risk estimate. Regardless of the algorithm used, it is currently unknown whether their implementation in AS patients is successful.

As many aspects on CV risk, its risk factors, and management are still unclear, we 1) assessed the prevalence of CV risk factors as compared to the prevalence of these risk factors in the general Dutch population; 2) estimated the 10-years CV risk in AS patients according to CV risk algorithms used in the Dutch, European and American CV-RM guidelines and 3) investigated whether AS patients at increased CV risk receive optimal preventive treatment according to the current Dutch CV-RM guideline in a large cohort of AS patients with active disease.

## Methods

### Study population

This cross-sectional study was conducted retrospectively. The study population consisted of 254 consecutive AS patients who were recruited from two simultaneously running observational prospective cohorts at the rheumatology department of the Jan van Breemen Research Institute | Reade, Amsterdam from August 2004 till August 2012. All patients fulfilled the 1984 Modified New York criteria for AS [13]. TNF-α blocking therapy naïve patients were included when they were eligible for TNF-α blocking therapy according to the Dutch consensus statement on the initiation of TNF-α blocking therapy in AS [14]. Approval was obtained from the local ethics committee (Ethics Committee of the Slotervaart Hospital and Reade, Amsterdam, The Netherlands) and all participating patients gave written informed consent.

### Patient characteristics

All participating patients underwent a physical examination and an interview to record details about disease history, clinical characteristics and demographics before start of TNF-α blocking therapy. Special attention was paid to hypercholesterolemia, hypertension, overweight, type 2 diabetes mellitus (DM), and smoking. History of cardiovascular disease (CVD) was assessed, including coronary heart disease, congestive heart failure, cerebrovascular disease and peripheral arterial disease. The use of cholesterol lowering agents, antihypertensives and non-steroidal anti-inflammatory drugs (NSAIDs) was also recorded. Disease activity was measured with the Bath Ankylosing Spondylitis Disease Activity Index (BASDAI). Physical examination included height, weight and blood pressure measurements. Blood pressure was measured manually according to the standard hospital procedures. Blood sample measurements (non-fasting) included standard hematological assessment, C-reactive protein (CRP), erythrocyte sedimentation rate (ESR), creatinine and cholesterol levels. A single laboratory analyzed all blood samples.

### General population

Prevalences of CV risk factors in this cohort were compared to data from the general Dutch background population from TNS-NIPO [15] or the Dutch National Institute for public health and environment (RIVM) [16]. The same definitions for the hypertension, hypercholesterolemia and overweight for the background population and included AS patients were used. Overweight was defined as a BMI ≥25 kg/m^2^ and obesity as a BMI ≥30 kg/m^2^. Hypertension was defined as a systolic blood pressure (SBP) ≥140 mmHg and/or a diastolic blood pressure (DBP) ≥90 mmHg and/or the use of antihypertensive drugs. Hypercholesterolemia was defined as total cholesterol (TC) level of ≥6.5 mmol/L and/or use of cholesterol lowering drugs.

### CV risk assessment

The CV risk stratification methods of the Dutch, European and American CV-RM guidelines were used to estimate 10-years CV risk [7,8,17]. To adjust for the increased CV risk in AS patients we also calculated the 10-years CV risk by adding 15 years to the age of the AS patients in the Dutch risk stratification method. This is standard practice in all Dutch patients with RA and/or DM, as the CARRE study by Van Halm et al. showed that the CV risk of RA patients equals that of DM patients [17,18].

### CV risk management

The Dutch CV-RM guidelines were also applied to investigate whether AS patients at increased CV risk received adequate preventive treatment [18]. The Dutch CV-RM charts were used, as they are specific for the Dutch population and assesses the total CV burden instead of CV mortality alone. In the Dutch CV-RM guidelines, gender, age, smoking status, SBP and the TC/HDL-C ratio are used to calculate CV risk. Smoking was defined as currently smoking at least once per week. Patients for whom no cholesterol levels or blood pressure measurements were available, patients with previous CVD and patients younger than 40 were excluded, as the Dutch CV-RM guidelines are not validated for patients aged below 40 years. We categorized patients into three groups: (1) patients who had an indication for preventive treatment but did not receive this; (2) patients who were inadequately treated as they did not meet the treatment goals (i.e. a SBP ≤140 mmHg and/or an low density lipoprotein (LDL) -cholesterol ≤ 2.5 mmol/L); (3) patients who were treated adequately or had no increased CV risk or CV risk factors.

### Statistical analysis

For data analysis SPSS Version 21.0 (SPSS, Chicago, Illinois, USA) was used. Values are expressed as mean ± standard deviation (SD), median (interquartile range) (IQR) or percentages, as appropriate. For comparisons of variables with a normal distribution between two groups, independent *t*-tests were used. The Pearson’s chi-square test was performed on dichotomous variables.

## Results

### Basic demographics and disease characteristics

In total, 254 consecutive AS patients were included (Table 1). The median age of the total AS population was 42 years (IQR 35–51), and 170 patients (67%) were male. Median disease duration was 7 years (IQR 2–14), median CRP was 8 mg/l (IQR 3–25) and mean BASDAI score was 5.9 ± 1.9. One-hundred-and-eighty patients (71%) used NSAIDs. SBP and DPB levels did not significantly differ between patients using NSAIDs (mean SBP/DBP 126/80 mmHg) and not using NSAIDs (mean SBP/DBP 131/82 mmHg), p = 0.4 and p = 0.9 respectively. However, patients who used NSAIDs, as compared to those who did not, significantly more often used antihypertensive medication (26% vs. 9%, p = 0.003).

**Table 1:** **Patient characteristics N = 254**

**Demographic**	
Age, years (median, IQR)	42 (35–51)
Male (number, percentage)	170 (67%)
**Disease status**	
Disease duration, years (median, IQR)	7 (2–10)
HLA-B27 positive (number, percentage)	196 (77%)
C-reactive protein, mg/l (median, IQR)	8 (3–25)
Erythrocyte sedimentation rate, mm/h (median, IQR)	19 (7–37)
BASDAI (0–10) (mean, SD)	5.9 ± 1.9
**CV risk factors**	
Prior CVD (number, percentage)	8 (3%)
Myocardial infarction (number)	4
TIA/CVA (number)	4
Peripheral arterial disease (number)	0
Smoking (number, percentage)	109 (43%)
Systolic blood pressure, mmHg (mean, SD)	127 ± 17
Diastolic blood pressure, mmHg (mean, SD)	81 ± 10
Hypertension (SBP ≥ 140 and/or DBP ≥ 90 and/or antihypertensive drugs) (number, percentage)	81 (32%)
Hypercholesterolemia (TC ≥ 6,5 mmol/L and/or cholesterol lowering drugs) (number, percentage)	30 (12%)
Diabetes type 2 (number, percentage)	5 (2%)
Body mass index, kg/m2 (mean, SD)	26.2 ± 4.6
Overweight (≥25 BMI) (number, percentage)	134 (53%)
Kidney function (eGRF Cockcroft Gault) ml/min (mean, SD)	125.5 ± 34.2
**Lipid levels**	
Total cholesterol (mmol/l) (mean, SD)	4.96 ± 0.94
Triglycerides (mmol/l) (median, IQR)	1.28 (0.92-1.87)
HDL cholesterol (mmol/l) (mean, SD)	1.32 ± 0.39
LDL cholesterol (mmol/l) (mean, SD)	2.92 ± 0.81
Total cholesterol/HDL-C ratio (mean, SD)	4.05 ± 1.39
**Medication**	
Statins (number, percentage)	16 (6%)
Antihypertensive drugs (number, percentage)	36 (14%)
NSAIDs (number, percentage)	180 (71%)
Diclofenac (number, percentage)	46 (26%)
Etoricoxib (number, percentage)	41 (23%)
Naproxen (number, percentage)	29 (16%)
Ibuprofen (number, percentage)	21 (12%)
Other (number, percentage)	43 (23%)

Data is expressed as number (percentage), mean ± standard deviation or median (range) as appropriate. BASDAI: Bath Ankylosing Spondylitis Disease Activity Index, CVD: Cardiovascular Disease, TIA: Transient Ischemic Attack, CVA: Cerebrovascular Accident, SBP: Systolic Blood Pressure, DBP: Diastolic Blood Pressure, BMI: Body Mass Index, eGRF: estimated Glomerular Filtration Rate, TC: Total Cholesterol, HDL: High Density Lipoprotein, LDL: Low Density Lipoprotein, NSAID: Non-Steroidal Anti-Inflammatory Drug.

### Prevalence of CV risk factors

**Figure 1:**
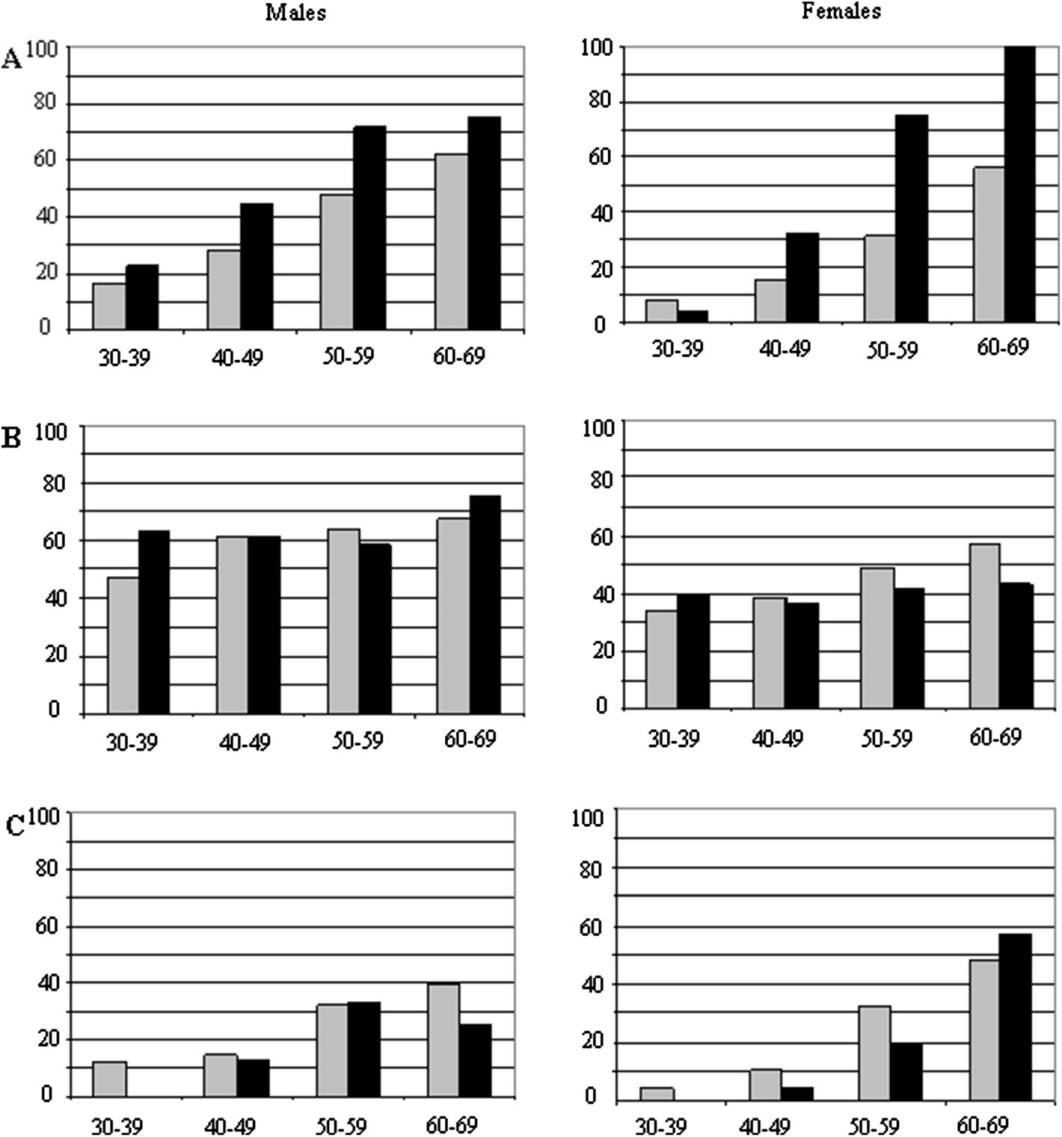
**Prevalences of hypertension (A), overweight (B) and hypercholesterolemia (C) in AS patients compared to the general Dutch population in four different age categories.** Legend: X-axis: age categories, Y-axis: percentage, black: ankylosing spondylitis population, grey: general Dutch population.

Of 254 AS patients, eight patients (3%) had a history of CVD and five patients (2%) were diagnosed with type 2 DM. One-hundred-and-nine patients (43%) smoked (45% of the men and 38% of the women) compared to 27% of the general Dutch population (30% of the men and 24% of the women) p = 0.001. Mean BMI was 26.2 ± 4.6, 134 patients (53%) were overweight and 44 patients (17%) were obese. Hypertension was present in 103 AS patients (41%) versus 31% in the general population (p = 0.026). Hypercholesterolemia was present in 30 patients (12%). The results for hypertension, hypercholesterolemia and overweight are displayed categorized by age and gender in Figure 1. Compared to the general Dutch population, the prevalences of hypertension and smoking are higher in AS patients, the latter, however, only in AS males.

### CV risk assessment

Out of 254 patients, CV risk according to the Dutch CV-RM guidelines could be assessed in 130 patients (Table 2). One-hundred-and-eight patients were excluded as they were younger than 40 years, eight patients due to a history of CVD, and eight patients due to lack of cholesterol or blood pressure data. According to the Dutch CV-RM guidelines, nine patients (7%) were at high CV risk, sixteen patients (12%) were at intermediate CV risk and 105 patients (81%) were at low CV risk. When adding 15 years to the age of AS patients, CV risk could be assessed in 231 AS patients (excluding seven patients due to age below 40 years, eight patients due to CVD history, eight patients due to lack of data). The percentage of high CV risk patients increased to 26% (Table 3). For the European and American guidelines the percentage of patients at high CV risk was 22% and 29%, respectively.

**Table 2:** **Patients at risk according to Dutch CV-RM guideline** r**isk scores and treatment**

**Dutch CV-RM guidelines**	**Total N = 254**	**Indication for CV risk medication N = 51**	**Inadequately or not treated N = 39**	**Statins N = 7**	**Antihypertensive drugs N = 27**	**Both statin and antihypertensive N = 9**
High risk >20%	9	9	9	2	2	1
Intermediate risk 10-20%	16	10	8	1	6	1
Low risk <10%	105	24	14	1	16	5
Secondary CV prevention	8	8	8	3	3	2
CV risk not determined*	116	N.A.	N.A.	0	0	0

NA; not applicable N; number of patients, CV; cardiovascular * Patients who could not be included because of age <40 years or missing cholesterol/blood pressure values.

**Table 3:** **Cardiovascular risk according to Dutch, European and American guidelines**

**Total number of patients**	**USA n = 130**	**EUR n = 130**	**DUTCH n = 130**	**DUTCH + 15 yr n = 231**
High CV risk	29% (>7,5%)	22% (>5%)	7% (>20%)	26% (>20%)
Medium CV risk	13% (5–7,5%)	63% (1-5%)	12% (10-20%)	15% (10-20%)
Low CV risk	58% (<5,0%)	15% (<1%)	81% (<10%)	59% (<10%)

USA = American; EUR = European; N; number of patients, CV; cardiovascular. Between brackets the cutoff values for high, intermediate and low risk of a vascular event for the appropriate cardiovascular risk management guidelines.

### CV risk management

According to the Dutch CV-RM guidelines, of the 130 screened AS patients, nine patients (7%) were not treated at all while there was an indication for primary CV risk prevention treatment and 22 patients (17%) were inadequately treated, as treatment targets for blood pressure or cholesterol levels were not reached (Table 2). Of the eight patients with a history of CVD, all received secondary prevention treatment, however, treatment targets were not reached in any of them.

**Figure 2:**
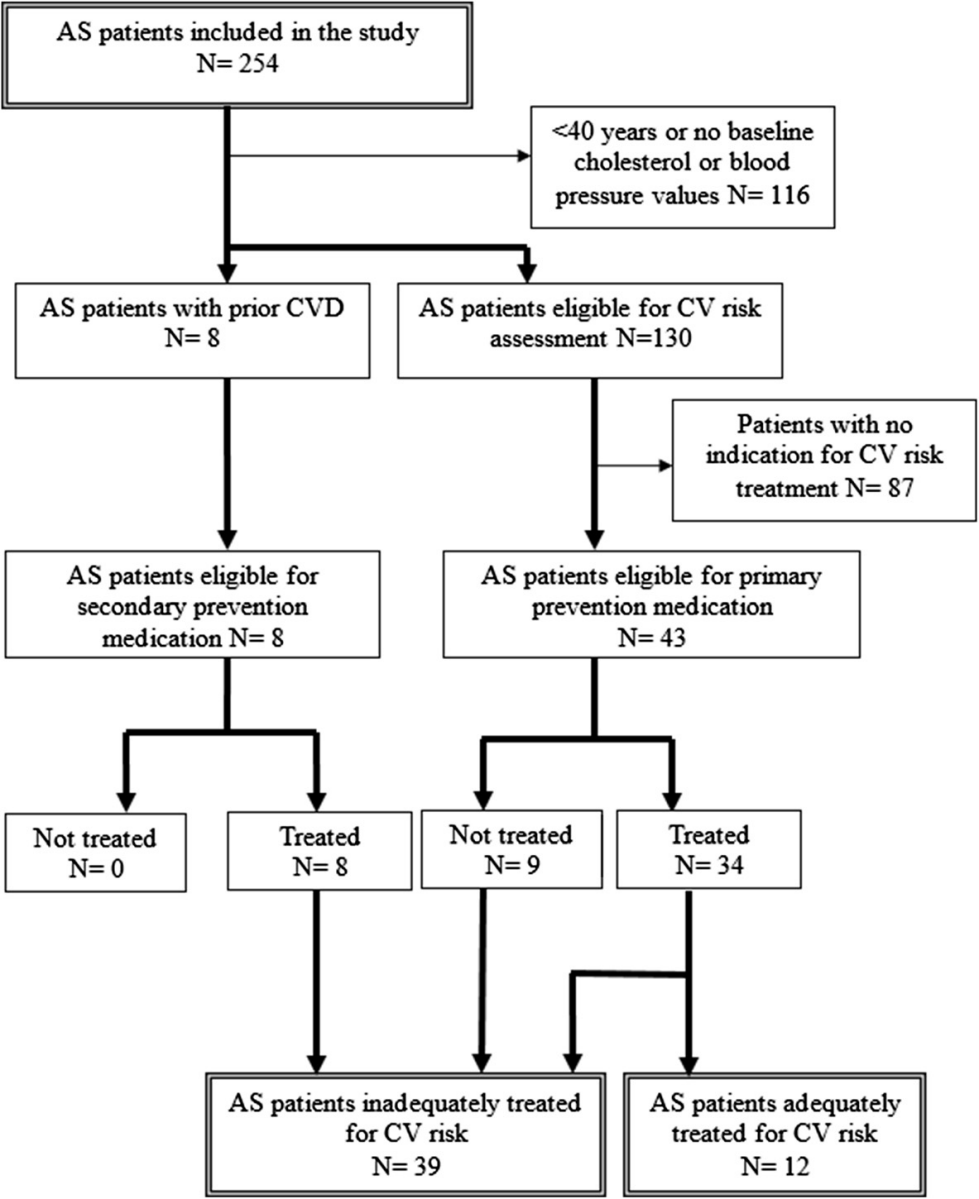
**The identification of AS patients at increased CV risk.** Legend: AS; ankylosing spondylitis, CV; cardiovascular, CVD; cardiovascular disease, n; number of patients. Cardiovascular risk treatment is according to the Dutch cardiovascular risk management guideline. Inadequately treated: not treated with statins and/or antihypertensive medication while there was an indication for primary or secondary CV risk prevention treatment not meeting treatment goals.

In total, of the 138 AS assessed patients, 51 patients had an indication for CV risk treatment of which 42 patients (82%) received some form of CV risk medication (Figure 2). However, 39 (76%) of the 51 patients were treated inadequately due to failure to reach treatment targets for hypertension or hypercholesterolemia or due to total lack of CV risk medication (Table 2). When the modification factor (adding 15 years) was applied, undertreatment of CV risk management was present in 44% of all patients.

## Discussion

In this observational cohort study we observed increased rates of hypertension and smoking in patients with active AS when compared to the general Dutch background population. This observation together with a documented elevated CV risk in AS patients emphasizes the need for optimal CV-RM. The majority of patients at increased CV risk received treatment, but CV-RM remains an important challenge for treating physicians, as treatment targets were often not achieved.

Available studies of the prevalence of CV risk factors in AS patients yield varying results. These differences could be explained by the heterogeneity of study populations investigated [4-6,19]. Given the close relationship between inflammation, CV risk factors, and CV risk, we choose to assess CV risk and its risk factors in AS patients with high disease activity. In this selected population, we observed higher prevalence rates of hypertension and smoking, but not hypercholesterolemia and overweight. Inflammation may deteriorate CV risk factors, but patients with active disease may also experience more pain and physical stiffness, which can induce physical inactivity, increasing the chance of overweight and hypertension. The common use of NSAIDs might also contribute to the increased prevalence of hypertension. In fact, although the blood pressure was not significantly different, anti-hypertensive agents were prescribed significantly more in AS patients on NSAIDs.

The number of patients at high CV risk varied widely depending on the CV risk algorithm that was used. According to the Dutch CV-RM guidelines, 7% of assessed AS patients was at high CV risk, while 22% and 29% were at high risk according to either the European or American CV-RM guidelines. Overall, 5% of AS patients was at high CV risk according to all three guidelines. The large variation between the different CV-RM guidelines is remarkable. Several factors can explain this variation. First, the clinical outcomes on which the different guidelines are based vary from 10-years CV risk to develop a fatal atherosclerotic CV event (European CV-RM guidelines), 10-years CV risk to develop a fatal or non-fatal myocardial infarction or stroke (2013 ACC/AHA guidelines), or to 10-years CV risk to develop a fatal or non-fatal atherosclerotic event (Dutch CV-RM guidelines) [7,8]. Second, all guidelines are applicable for different age categories, i.e. Dutch for 40–70, European for 40–65, and ACC/AHA for 40–79 years of age.

To date, it is recognized that systemic inflammation is an important CV risk factor. However, none of the CV-RM guidelines adjust for the inflammatory burden in AS patients, which could lead to underestimation of CV risk [7,8]. The EULAR task force advised to use a multiplication factor of 1.5 to CV risk to adjust for the increased CV risk in RA when patients have a disease duration >10 years, rheumatoid factor or anti-cyclic citrullinated peptide positivity, or presence of extra-articular manifestations [12]. The Dutch CV-RM guidelines add 15 years to the age of all RA patients to calculate CV risk, as is also applied in DM patients [18]. Currently, due to lack of evidence, it is unknown if, and if yes, what modification factor for CV risk assessment in AS patients is most appropriate. To investigate the impact of a modification factor for the increased CV risk in AS patients, we used the same modification factors as used in RA, for our group of AS patients. When adding 15 years to the age of the AS patients in this cohort, the percentage of patients with high CV risk increases from 7% to 26%. Subsequently, undertreatment of this CV risk increased from 28% to 44%. Future studies should be conducted to investigate which CV risk stratification method most accurately predicts CV risk in AS patients.

Since most patients with AS die from complications of atherosclerosis, early identification and optimal CV-RM is of utmost importance. It is reassuring that the awareness of CV-RM was good, as the vast majority of AS patients at risk for CV disease received treatment. However, it is alarming to see that in 76% CV-RM was suboptimal, as treatment targets were not reached. Moreover, in none of AS patients needing secondary prevention of CV disease, treatment targets were reached. Undertreatment of CV risk (factors) can be caused by multiple factors. First, there might be a lack of awareness of the increased CV risk in AS patients in treating physicians. Second, CV-RM, including CV risk screening, treatment, and follow-up, is time consuming and lack of time to act on guidelines presents an additional hurdle. Also, it is not clear who has responsibility for CV-RM in AS patients, as this can be the primary care physician, the treating rheumatologist, or both. The number and complexity of available guidelines also impede their implementation. Finally, since AS is often diagnosed early in life, implementing a lifetime treatment with medication for CV risk (factors) is probably challenging for the physician and is prone for non-compliance. These challenges need to be addressed to achieve a decrease in CV morbidity and mortality in AS patients. It is therefore important that CV-RM in AS patients should be a joint effort for the primary care physician and treating rheumatologist. We should strive for increased awareness of CV risk and need to identify and target high-risk individuals for primary and secondary prevention.

A major strength of this study is the in-depth evaluation of CV risk and CV risk factors. We estimated CV risk according to various CV-RM assessment tools, thereby giving an overview of the different outcomes on CV risk in this AS population. However, only AS patients with active disease and from a single rheumatology centre were included which hampers generalizability. Finally, due to the retrospective design of this study, there was no data available on the reasons why CV risk treatment was suboptimal. This complicates further evaluation of CV-RM implementation, as it is possible that increased CV risk is acknowledged but treatment is not initiated because of side effects, comorbidity, or patients’ non-adherence.

## Conclusions

In conclusion, this study shows that the prevalence of hypertension and smoking is increased in AS patients with active disease. Also, AS patients at increased CV risk received medication, but treatment targets were often not achieved. Our data illustrate the need for programs to improve the quality of CV-RM in AS.
